# Help-seeking behavior among college students with suicidal ideation: barriers, facilitators, and social actors

**DOI:** 10.1186/s41155-025-00374-x

**Published:** 2026-01-22

**Authors:** Hareli Fernanda Garcia Cecchin, Sheila Giardini Murta

**Affiliations:** 1 Universidade Federal do Tocantins – UFT, Palmas-TO, Brasil; 2 Universidade de Brasília – UnB Instituto de Psicologia - IP, Brasília-DF, Brasil; 3 Pró-reitoria de Assuntos Estudantis – PROEST, Quadra 109 norte, Av. NS 15, ALCNO-14, Bloco da reitoria, térreo, Plano diretor norte, Palmas-TO, CEP 77001-090 Brasil

**Keywords:** Help-seeking behavior, Suicide prevention, Mental health literacy, Higher education, Mixed-methods, Institutional support, Stigma

## Abstract

**Background:**

Suicide among university students is a serious public health concern, often exacerbated by academic pressure, mental health stigma, and limited institutional support. Understanding contextual factors is key to developing effective prevention strategies.

**Objective:**

This study aimed to analyze the barriers and facilitators of help-seeking behavior among college students with suicidal ideation and to identify the social actors involved in help-seeking.

**Methods:**

A mixed methods approach was adopted, integrating qualitative and quantitative data to obtain a comprehensive understanding of help-seeking behavior. Qualitative data was collected through interviews with 20 students and 12 course coordinators, and a focus group with 6 administrative staff members. Quantitative data were obtained from a questionnaire completed by 22 mental health professionals, which included both multiple-choice and open-ended questions. The integration of findings occurred during the interpretation phase, allowing the quantitative results to complement and expand the qualitative analysis. Content Analysis and simple descriptive statistics were used.

**Results:**

Seventeen barriers and twelve facilitators of professional help-seeking were identified and organized across individual, interpersonal, organizational, and social levels. The main barriers included low perceived need for professional care, self-stigma, emotional avoidance, insufficient social support, and institutional fragility. Facilitators comprised mental health literacy, encouragement from close individuals, previous positive experiences with therapy, financial aid for mental health treatment, and having accessible on-campus services. Professors, peers, and administrative staff emerged as key social actors in referring students to support services, highlighting the relevance of peer and faculty networks in suicide prevention.

**Conclusion:**

Low perceived need, self-stigma, and institutional barriers significantly hinder help-seeking among university students with suicidal ideation. Conversely, mental health literacy, social support, and institutional engagement foster professional help-seeking and trust in mental health care. These findings underscore the need for integrated, context-sensitive university strategies that combine psychoeducational interventions, gatekeeper training, and structural support to promote a culture of early intervention and belonging. The study contributes to suicide prevention efforts in higher education by emphasizing that collective, equity-oriented approaches are more recommended than exclusively individual interventions.

**Supplementary Information:**

The online version contains supplementary material available at 10.1186/s41155-025-00374-x.

## Introduction

Mental health difficulties, such as anxiety and depression, often emerge when young people begin their university studies (Mortier et al., 2018). In Brazil, national surveys indicate that psychological distress is widespread among undergraduates, with anxiety, discouragement, and sleep disturbances among the most frequent complaints (Fonaprace, 2019). Some cases progress to suicidal ideation, illustrating the magnitude of this public health concern. Institutional reports also show that episodes of student suicide have been documented in most Brazilian Federal Universities (Araújo, 2020), highlighting the need for integrated mental health actions within higher education.

Suicide prevention requires a multifaceted approach that combines early intervention, restriction of means, and the promotion of life skills among young people (World Health Organization, 2014). According to this framework, access to mental health care and the willingness of individuals to seek professional help are equally important. However, data from the same national survey reveal that most students have never sought psychological care (67.6%), with even lower rates observed in the North region of the country (79.7%) (Fórum Nacional de Pró-Reitores de Assuntos Estudantis, 2019). These findings point the importance of understanding the contextual and institutional factors that influence help-seeking behavior among university students.

As a result, many studies have focused on the understanding the barriers and facilitators for help-seeking behavior in students. The main barriers that prevent students with suicidal ideation from seeking mental health services are: stigma, low perceived need, difficulty in identifying mental health symptoms, preference for self-management of problems, and tendency towards self-sufficiency (postponing until symptoms become uncontrollable) (Czyz et al., 2013; De Luca et al., 2020; Downs et al., 2012; Eisenberg et al., 2012; Ibrahim et al., 2019; Lueck et al., 2020; Sagar- Ouriaghli et al., 2020). Perceived need is the perception that professional help is not necessary due to minor or transitory problems (Czyz et al., 2013).

Stigma has been consistently identified as one of the most significant barriers to mental health help-seeking among university students (Eisenberg et al., 2012). In this study, stigma was understood according to the multidimensional model proposed by Corrigan and Watson (2002), which differentiates between public stigma – the set of negative social stereotypes directed toward individuals with mental health problems – and self-stigma, referring to the internalization of these negative beliefs by the person affected. In higher education settings, self-stigma is particularly relevant, as it reduces students’ sense of self-efficacy and discourages them from seeking psychological help (Vogel et al., 2007; Eisenberg et al., 2012). Understanding stigma from this theoretical perspective enables a more comprehensive analysis of how individual beliefs and social norms interact to shape help-seeking behaviors in higher education settings.

On the other hand, there are facilitators for seeking help. The most cited in the literature are: encouragement from close people such as family and friends, understanding the symptoms of mental disorders and the need to seek help (mental health literacy), positive beliefs about treatment, availability of services (extended opening hours, among others) and having close friends or family members who have used treatment (Downs et al., 2012; Eisenberg et al., 2012; Ibrahim et al., 2019; Sagar- Ouriaghli et al., 2020).

Help-seeking behavior has been the focus of several suicide prevention interventions, since the chance of completed suicide drops drastically in individuals who receive adequate treatment (Albuquerque et al., 2020). A recent systematic literature review demonstrated that help-seeking behavior is the main target of suicide prevention programs, whether through the provision of information, psychoeducation, or training of gatekeepers to welcome and refer students to health services (hidden to preserve peer review). Studying the factors that promote or hinder help-seeking in mental health is essential to develop effective strategies that reduce barriers and increase access to appropriate care, directly contributing to suicide prevention and promoting the well-being of university students.

Identifying contextual factors that shape help-seeking behavior is a crucial step toward designing culturally sensitive suicide prevention programs. This study is part of a broader project focused on developing a suicide prevention program for college students, guided by the Intervention Mapping model (Bartholomew- Eldredge et al., 2016; Kok et al., 2017; Peters, 2014). Accordingly, this study sought to analyze the barriers, facilitators, and social actors influencing professional help-seeking among university students with suicidal ideation in Northern Brazil.

## Method

The study followed a sequential exploratory design, with multiple informants and a data triangulation strategy (Creswell, 2010), using a mixed-method approach guided by the Intervention Mapping model (Bartholomew- Eldredge et al., 2016). The study included four phases of data collection and analysis, in which one stage supported the next. Subsequently, the results of both phases were integrated during the interpretation phase. This design was used to obtain a clearer and more comprehensive understanding of the help-seeking behavior of college students. The study was organized using the Mixed Methods Article Reporting Standards (MMARS) (Levitt et al., 2018).

The study was carried out at a public university in the northern region of Brazil, which offers undergraduate and postgraduate courses in various fields of knowledge. The university operates in 5 municipalities, comprising 5 campuses. This research is part of a broader project on university students’ mental health and suicidal behavior conducted in Brazilian higher education institutions. Previous studies from the same project examined risk factors (hidden to preserve peer review) and protective factors (hidden to preserve peer review) associated with suicidal ideation. The present article focuses on the phase addressing barriers, facilitators, and social actors involved in help-seeking behavior. Together, these studies contribute to a comprehensive understanding of suicide prevention among university students in Brazil.

### Participants

The study involved four groups of participants: (a) undergraduate students with suicidal ideation, (b) mental health professionals working in student support services, (c) course coordinators, and (d) technical-administrative staff. In total, 20 undergraduate students, 22 health professionals, 12 undergraduate course coordinators, and 6 technical-administrative staff participated.

Students were identified through the university’s mental health support program, which monitors cases of students receiving psychological or psychiatric care outside the institution. These students sought treatment from private health professionals and paid for these services using a financial subsidy provided by the university to facilitate access to mental health care.

The sample was defined based on information from licensed psychologists and physicians who had previously evaluated the students. Health professionals, including psychologists and physicians, conducted initial assessments of students who presented with mental health concerns. The diagnosis of suicidal ideation was based on standardized clinical interviews conducted by licensed mental health professionals. These interviews were guided by established diagnostic criteria from the Diagnostic and Statistical Manual of Mental Disorders (DSM-5). Key indicators for the diagnosis of suicidal ideation included the presence of thoughts about self-harm or suicide, reported plans or means for committing suicide, and any previous suicide attempts.

The research team did not conduct any diagnostic procedures; instead, it relied on institutional records documenting that students had been clinically assessed and met the inclusion criterion of suicidal ideation. This approach ensured that participant selection was based on verified clinical information. The final sample consisted of students who met the inclusion criteria of being diagnosed with suicidal ideation and being from a low-income family. These students had received mental health treatment for 12 months and had documented suicidal ideation.

Sociodemographic information, including age, gender, and race/ethnicity, was collected only for the student group, which represented the primary target population of the study. For the other participant groups – mental health professionals, course coordinators, and administrative staff – demographic data were limited to gender, age, and professional role. This decision was made to preserve participant confidentiality in small institutional contexts, as the analysis of racial and ethnic variables was not central to the study objectives for these groups, which were included primarily to enable data triangulation across institutional perspectives.

### Instruments

The instruments (interviews, focus group and questionnaire) were developed based on research questions and literature (Bartholomew-Eldredge et al., 2016), described in Table 1. Both instruments aimed to explore barriers, facilitators, and social actors related to students’ help-seeking behavior for mental health problems.

**Table 1:** Data collection instruments used with each audience

Public	Instruments	Questions
Students	Individual interview	• What was it like for you to ask for help? • Could you tell us how it went? • Did someone refer you or did you look for it yourself?
Technical-administrative staff	Focus group	• How did cases of students with suicidal behavior come to you: spontaneous demand, referral or active search? • In cases where they were referred by someone, who referred them the most: students, technicians or staff? • When a student needs help with a mental health problem at university, is it easy or difficult to ask for help? Why? • What inhibits help-seeking among university students? What facilitates help-seeking?
Health Professionals	Questionnaire	• What factor(s) make it difficult for students to seek help from a healthcare professional? • What factor(s) make it easier for students to feel motivated to seek help from a health professional? • Who are the people the student most often asks for help, or who has helped him/her the most, before seeking professional help?
Course Coordinators	Individual interview	• How do you perceive the opportunities that the university offers for students to ask for help when they have a mental health problem? • How do you perceive the barriers that the university offers to students asking for help when they have a mental health problem?

The interview and focus group guides were semi-structured and organized around three thematic axes: (1) barriers to help-seeking, (2) facilitators and social support networks, and (3) social actors related to students’ help-seeking behavior for mental health problems. Each topic included guiding questions and probes designed to elicit detailed narratives. Examples of probes included: “Can you tell me more about what made it difficult to seek help?”, “How did others respond when you shared your situation?”, and “What kind of support did you expect from the university?”. The interviewer used prompts to explore emotional, relational, and contextual aspects of help-seeking, encouraging participants to describe specific experiences and perceptions. The guide was developed by the first author, reviewed by one senior researcher in mental health, and refined after a pilot interview with a student to ensure clarity and sensitivity to the topic. A complete version of the interview and focus group outlines is provided in Table 1 in Additional file 1.

The focus group followed a semi-structured format, guided by open-ended questions designed to elicit participants’ perceptions and experiences related to help-seeking. Although an initial question guide was developed (e.g., “When a student needs help with a mental health problem at university, is it easy or difficult to ask for help? Why?”), the facilitator used these prompts flexibly, encouraging participants to elaborate freely on their feelings, attitudes, and contextual factors. This approach allowed for a conversational flow, enabling new themes to emerge spontaneously while ensuring that all core topics relevant to the study objectives were covered. This flexible strategy is consistent with qualitative research best practices for focus groups (Krueger & Casey, 2014).

The questions in the questionnaire sent to health professionals presented response options derived from the data obtained in the interviews with the students. The three questions, described in Table 1, allowed professionals to provide additional responses beyond those listed. All instruments were pilot tested with a small group of university staff and students to ensure clarity and relevance. Minor wording adjustments were made before data collection. Data collection was carried out by a researcher with a degree in Psychology, ten years of experience in qualitative research and familiarity with the institution’s culture.

### Data collection and procedures

After selecting participants through purposive sampling, data collection was carried out sequentially in four phases between May and August 2021. In Phase 1, individual interviews were conducted with 20 students who received financial aid for mental health treatment. In Phase 2, three biweekly focus group sessions were held with six technical-administrative staff members working in student assistance and academic support services. In Phase 3, a structured online questionnaire was sent to 41 mental health professionals who had provided care to the aforementioned students; 22 responded (53.6%). Finally, in Phase 4, 12 course coordinators were interviewed individually. This stepwise process allowed the qualitative and quantitative data to inform each other and ensured a comprehensive understanding of help-seeking behavior within the institutional context.

For the group of undergraduate students, inclusion criteria were: (a) being enrolled at the university at the time of data collection, (b) having been clinically diagnosed with suicidal ideation by a licensed mental health professional, and (c) receiving financial aid from the university to support mental health treatment. Exclusion criteria included students whose records indicated recent suicide attempts requiring hospitalization or interruption of treatment. Among the 118 students registered in the university’s financial aid program for mental health treatment, 50 had a documented diagnosis of suicidal ideation. From this subgroup, 20 students were selected to ensure an even distribution across the five university campuses and to prioritize those who had been receiving financial aid for the longest period. At the time of the interviews, all participants were in remission from suicidal ideation and under ongoing clinical follow-up, ensuring their psychological safety to participate.

It is important to note that the institutional records used to identify eligible students referred to the 2020 academic year, which was the most recent period with complete information on student mental health assistance. Therefore, although the institutional data correspond to 2020, all interviews, focus groups, and questionnaires were conducted in 2021. This temporal distinction ensured that participants had received or were still receiving mental health support during the period documented in the university’s records. Although this coincided with the COVID-19 pandemic, the study was not designed to specifically assess the pandemic’s effects on mental health or help-seeking behavior. However, this context is acknowledged as potentially influencing students’ experiences and perceptions.

Among the students interviewed, 16 were female and 4 were male (see Table 2). Students were recruited via email and invited to an individual videoconference interview at a date and time convenient for the participant. Interviews lasted from 17 to 50 min, with an average time of 28 min. All invited students received an explanation of the study objectives, procedures, and confidentiality measures and provided informed consent electronically before the interviews.

**Table 2:** Data collection instruments used with each audience

Name	Gender	Sexual orientation	Age	Ethnicity	Course	Course modality
Participant1	Female	>Heterosexual	>23	Biracial (Black-White)	Forestry Engineering	Integral
Participant2	Female	Heterosexual	23	Black	Bioprocess Engineering	Integral
Participant3	Male	Heterosexual	24	Black	Lyrics	Morning
Participant4	Female	Heterosexual	24	Biracial (Black-White)	Psychology	Integral
Participant5	Male	Heterosexual	28	White	Agronomy	Integral
Participant6	Female	Homosexual	23	Biracial (Black-White)	Psychology	Integral
Participant7	Male	Homosexual	24	Biracial (Black-White)	Pedagogy	Morning
Participant8	Female	Heterosexual	27	Black	Social Service	Morning
Participant9	Female	Homosexual	23	Black	Journalism	Morning
Participant10	Female	Heterosexual	25	Biracial (Black-White)	Environmental Engineering	Integral
Participant11	Female	Homosexual	25	Indigenous	Medicine	Integral
Participant12	Female	Homosexual	32	Black	Pedagogy	Morning
Participant13	Female	Heterosexual	23	White	Forestry Engineering	Integral
Participant14	Female	Heterosexual	21	Biracial (Black-White)	Bioprocess Engineering	Integral
Participant15	Male	Heterosexual	21	Biracial (Black-White)	Bioprocess Engineering	Integral
Participant16	Female	Heterosexual	25	Biracial (Black-White)	Agronomy	Integral
Participant17	Female	Bisexual	25	Black	Medicine	Integral
Participant18	Female	Heterosexual	25	Biracial (Black-White)	Psychology	Integral
Participant19	Female	Homosexual	23	Biracial (Black-White)	Psychology	Integral
Participant20	Female	Homosexual	24	Biracial (Black-White)	Social Service	Morning

The same database was used to recruit health professionals. There were 41 health professionals who cared for the aforementioned students. These health professionals were recruited via email and received a link to the questionnaire. Of these, 22 health professionals (53.6%) responded (see Table 3) and two declined to participate citing ethical reasons. Mental health professionals cared for 1 to 11 students (M = 1.63; SD = 2.06).

**Table 3:** Sociodemographic data of mental health professionals

Variable	Description	Profession		Total
		Physician	Psychologist	
Gender	Female	2 (12%)	14 (87%)	16 (100%)
	Male	4 (66%)	2 (33%)	6 (100%)
Total	----------------	6 (100%)	16 (100%)	22 (100%)

The 37 course coordinators were recruited by email and invited to participate in an individual videoconference interview and then asked to choose a convenient date and time. Of these, 12 course coordinators (32.5%) responded (see Table 4).

**Table 4:** Sociodemographic data of course coordinators

Name	Gender	Course	Educational level	Working time
Participant 1	Male	Environmental Engineering	PhD	8
Participant 2	Male	Civil Engineering	PhD	8
Participant 3	Female	Right	PhD	7
Participant 4	Male	Physical education	Master's degree	0.5
Participant5	Female	Environmental Engineering	PhD	3
Participant 6	Female	Nutrition	PhD	8
Participant7	Female	Social Service	PhD	10
Participant 8	Female	Law	Master's degree	3
Participant 9	Female	Physical education	Master's degree	4
Participant 10	Female	Journalism	PhD	17
Participant 11	Female	Pedagogy	PhD	8
Participant 12	Male	Administration	PhD	3

Note: working time is expressed in years and refers to the time worked at the institution

The six technical-administrative staff members were selected based on convenience and their placement in a Student Assistance Center (SAC), the Pro-Rectory of Student Affairs (PROSA), the Pro-Rectory of Undergraduate Studies (PROUS) or the NZ Program 1. The staff members were recruited by e-mail, requesting one participant per department. Six of the eight invitations were responded to (see Table 5).

**Table 5:** Sociodemographic data of technical-administrative staff

Name	Gender	Training	Educational level	Workplace
Participant 1	Female	Psychology	PhD	SAC
Participant 2	Female	Psychology	Postgraduate studies	SAC
Participant3	Female	Psychology	Postgraduate studies	SAC
Participant 4	Female	Psychology	Postgraduate studies	SAC
Participant 5	Female	Social Service	Master's degree	SAC
Participant 6	Female	Social Service	Postgraduate studies	PROSA

### Data analysis

Data analysis was conducted in three complementary phases, following the sequential exploratory design of the study. First, data from each audience were analyzed individually. Interviews with students and coordinators were transcribed and analyzed using Content Analysis (Bardin, 2011), and the responses of health professionals to the questionnaire were analyzed using descriptive statistics. Focus group sessions were transcribed and analyzed using Content Analysis (Bardin, 2011), using the theme as a code and subsequent frequency count. Findings from the literature were taken into account, but there was flexibility to create new categories.

Second, the data from each audience were individually categorized according to the 5 dimensions of Intervention Mapping (individual, interpersonal, organizational, community, and social level). Third, the three databases were analyzed separately and subsequently grouped around large categories, inductively, according to the proximity of the themes. The combination of these codes also implied a new calculation of the data from health professionals, avoiding overlaps. A table was constructed that organized qualitative and quantitative data, allowing the comparison of results between multiple informants, following the recommendations of Levitt et al. (2018). The analysis focused on convergences and divergences.

The qualitative data were coded manually by the first author using inductive thematic procedures consistent with Bardin’s (2011) framework. Two researchers independently coded the material, compared their categorizations, and reached full consensus after discussion. Coding decisions and emerging categories were further refined through peer debriefing sessions with a senior researcher experienced in qualitative methods. Data organization, synthesis, and category mapping were conducted using Microsoft Word and Excel, allowing systematic tabulation and transparent traceability of codes, excerpts, and themes.

Member checking procedure, systematized by Birt et al. (2016), was used to assess the reliability of the qualitative data. A summary of the data was sent to students and course coordinators, who were asked to read and comment on whether the results described their experience. Participants were warned that reading the text could cause some discomfort and the contact details of the lead researcher were provided in case of need. No participant requested changes to the results.

All participants were duly informed about the research process and consented to participate by means of consent forms and a term of authorization for the use of images and sound. The study was approved by the Research Ethics Committee in Human and Social Sciences of the University of Brasília under opinion CAAE nº (hidden to allow peer review). The study was conducted in accordance with Resolution nº 510/2016 of the Brazilian National Health Council, which regulates research involving human subjects in the Human and Social Sciences (CNS, 2016). All participants consented to participate by means of the Free and Informed Consent Form (FICF).

## Results

### Barriers of help-seeking behavior

Data analysis identified 17 (seventeen) barriers to help-seeking behavior, illustrated in Table 6. The following responses were included in the questionnaire by health professionals: lack of financial means, racism experienced in previous therapy, difficulty in accessing financial aid from the university, lack of access to care or feeling that they will not be able to get it. In addition, some course coordinators (25%) mentioned that they did not see any barriers to seeking professional help at university.

**Table 6:** Barriers for professional help-seeking behavior according to students, health professionals, technical-administrative staff and undergraduate courses coordinators

Level	Barriers	Students	Health professionals	Technical	Coord
Individual	Tendency to self-sufficiency	25%	59.1%	---	8.33%
	Self-stigma	15%	40.9%	---	---
	Perceived need	30%	40.9%	---	8.33%
	Difficulty expressing emotions	5%	54.5%	---	---
	Questions about the effectiveness of the service	5%	18.2%	---	---
	Fear and embarrassment	5%	31.8%	---	8.33%
	Shame	15%	45.5%	---	8.33%
Interpersonal	Insufficient social support network	10%	31.8%	40%	42%
	Prefer to talk with friends	10%	27.3%	---	---
Organizational	Bureaucracy to obtain or renew financial aid	10%	4.54%	---	---
	Lack of professionals at the university	---	---	20%	25%
	Lack of publicity for the health service	15%	---	20%	41.66%
	Problems in the physical structure of the university’s mental health service	---	---	60%	---
	Problems in the provision of mental health services at the university	---	4.54%	40%	25%
Social	Racism experienced in previous therapy	---	4.54%	---	---
	Public stigma	10%	54.5%	40%	25%
	Lack of financial condition		9.09%	---	---

Note: Percentages are based on the total number of participants in each subgroup (*n* = 20 students, *n* = 22 mental health professionals, *n* = 12 coordinators, *n* = 6 administrative staff). Raw counts corresponding to these percentages were used in the analyses but are not displayed due to space constraints. Percentages do not add up to 100% because participants could mention more than one barrier to help-seeking behavior

*Tendency to self-sufficiency* category includes reports of students delaying seeking help until symptoms become unbearable. In some interviews, course coordinators mentioned that in some cases, students seek help from the university when they have no one else to turn to.

I think I reached the extreme level. Because I always wanted to be very strong, you know?! Always be very strong and endure things. And then I talked to my friends and tried to resolve things with myself until the moment that when I went to a teacher to ask for help, I was already extremely desolate, you know?! I was like, I already wanted to drop out of the course and go back home. (…) Today I understand, but at that time, I reached the extreme until I was able to go and ask for help (Student, P5).

In the category *self-stigma* are the reports of what the student will think about himself for undergoing mental health treatment.

I don’t know if it’s because of my upbringing, but it wasn’t easy for me, because I considered myself crazy, to reach the point of asking for help (Student, P12).

*Perceived need* encompasses the lack of perception of the severity of symptoms.

It’s hard to recognize that we need help, for me it’s a little hard to recognize that. However, I always asked the teachers: “When do I seek therapy?”, “When do I know that I can seek therapy?”, you know?! They said “When you can’t walk on your own two feet anymore”, when you can’t move anymore. Only I was able to move little by little. So I didn’t look for it, I felt a little difficulty, but I didn’t look for it (Student, P3).

The *difficulty of expressing emotions* covers reports of difficulty in identifying, dealing with and expressing emotions.

It was very difficult for me, especially talking about this subject, you know?! Because talking about this subject with the psychologist was very complicated. I had a lot of difficulty. Sometimes I don’t know how to say what I’m feeling, so I just stay quiet and avoid talking about it. It’s hard to express emotions. (Student, P9).

The category *questions about the effectiveness of the service* covers reports of not being certain that psychotherapy will be useful, because previous experience has not been satisfactory.

The psychologist also had a role in this. But there came a time when I had the impression that she was there simply, not to listen to me, but just to catch up on the conversations, you know?! She had a professional attitude, that she would pick up some little things there and then ask what the expressions were there, but it was very vague. I wanted to seek help later. But I kept thinking about seeking help so that I could continue in the same way, without feeling like I was actually being helped, so I took it and didn’t seek help anymore. And so, psychology, psychologists are a very good, respected field, I admire them a lot. But I wouldn’t consult any psychologist, particularly, because they are different paths and even though I know that you have to maintain impartiality, it’s impossible, right?! At some point or another, people end up saying something that they believe in, you know?! So for me, the only thing that would be useful would be someone from the church, precisely because they would be helping more in this area of faith (Student, P14).

The category *fear and embarrassment* include reports of fear of exposing oneself by seeking professional help, or fear of telling parents and disappointing them.

I believe that what sometimes makes it difficult for a student to ask for help is the same as what we are experiencing. It is not fear, it is exposing yourself to a problem that you did not want anyone to know about (Course coordinator, P27).

*Shame* encompasses reports of embarrassment in seeking professional help.

I think so. I don’t think so, I’m sure, that if I hadn’t sought help, I wouldn’t have talked to someone else. Because at the time I was very embarrassed to share, I didn’t share with friends or anything. (Student, P8).

The category *insufficient social support network* covers reports related to the difficulty of close people, such as parents, teachers and friends, in welcoming and supporting students with mental health problems. Among the course coordinators, this category includes reports of not supporting the student due to not knowing the mental health services on campus, having difficulty recognizing mental illnesses in students, being afraid to intervene and being held responsible later, lack of coordination between mental health services and course coordinators. According to technical-administrative staff, when students move away from their parents’ home and become distant from family and high school friends, they receive limited emotional support and guidance. They also lack close interaction with other students, which prevents them from recognizing the need for treatment or receiving advice from peers to seek help.

We have a huge percentage of students who are from out of town. It’s very rare for us to have this, I would say maybe 95% of our students are from out of town, which makes it even more difficult, because the contact with them is with their classmates where they live. They don’t have a family, their social support network is very limited and often limited to a context of not so deep relationships. So I think this also makes it difficult for them to realize that they have a serious problem, that they need help, and for other people to realize it and refer them (Technical-administrative employee, P36).

The category *preference for talking with friends* category covers reports that illustrate the preference for seeking help from family or friends and how this can delay or prevent seeking professional help.

No, it wasn’t easy. I always told people. But I told the wrong people. I think that’s what delayed the search, you know?! (Student, P10).

The category *Bureaucracy to obtaining or renew financial aid* includes reports of difficulties with the procedures necessary to obtain or renew the treatment subsidy offered by the university.

(…) but the university is very bureaucratic. I believe that not only it, but other universities (…). Because, I think that in the past, to get help we had to have a report first, and then, I think they changed the first appointment to be with the institution’s psychologist who could explain what was happening. But to continue we needed a report, right?! But that made me even more angry and upset (Student, P12).

The category *lack of professionals at the university* includes reports of a lack of professionals to meet demand and provide support to students.

I also think that sometimes there is a lack of personnel to work with this demand. When we think about work, we focus on our work in School Psychology, focused on the issue of learning. Mental health is a cross-cutting issue. So we end up taking actions to improve mental health. But then, if we focus on covering all the issues involving mental health, we won’t be able to cope. Because we also do this work of coordinating actions within the board, with teachers and students, everything that is there hindering the teaching-learning process of this student. So I keep thinking that there is a lack of hands to expand actions in this way (Technical-administrative employee, P39).

*Insufficient dissemination of information about available university services* was reflected in reports of students being unaware of existing resources, such as financial aid, the SAC, and mental health programs: “In fact, I didn’t know about the existence of (financial) aid at the time” (Student, P8).

The category *problems in the physical structure of the university’s mental health service* is made up of reports of problems in the physical structure of student assistance centers that do not allow accessibility for people with disabilities or privacy of care.

And I also think that the intervention space here is not very friendly, because we work… (…) it’s a large room with all the employees on either side of each other, a space with everything open. So the student comes in, to schedule an appointment with me, he has to go past the other employees. The entrance is visible, everyone sees him coming in, which is almost in front of the cafeteria and the leisure area they have. So everyone sees them coming in, and then there’s a room that is a little more, in quotation marks, isolated, because it’s only isolated by those simple partitions that you can hear. So I go to this room to attend to the student. Even so, no matter how hard you try to speak quietly, some of what you say escapes, so the environment is not very friendly. The physical structure, well, that’s it in short. The physical structure of the service *setting* is not the best possible, and we are in a better situation today, because two years ago we didn’t even have an individual service space, so it was much more complicated. When it was a very delicate issue, I had to go out and find an empty room to be able to assist the student in a more individualized context. Even so, it is still not ideal (Technical-administrative employee, P36).

The category *problems in the university’s mental health service* also consists of reports from course coordinators regarding delays in service and the absence of an online service channel, and from technical-administrative staff, who mentioned the lack of privacy for scheduling service and the student’s resistance to being welcomed by a social worker in student assistance sectors where there is no psychologist.

I came across this issue with the student whose parents had passed away. Because at first it comes to that discussion where one professor tells another, “A student lost her father, what do we do?” It comes about like that. And then I tried to make an official statement. (.) Despite having these opportunities on campus, it seems like they don’t interact much. They work together, but when you need, for example, a certain demand, it takes a while to happen. So, for example, I got the answer (…) after about 10 days of trying to get in touch. (…) So that takes a little more time. It seems like it didn’t flow with the certain urgency that the issue needed. I think that if we think about this, also, for a suicide situation, if it takes too long it could be fatal (Course coordinator, P25).

The category *public stigma* is made up of reports that refer to the thoughts and attitudes of others related to someone seeking professional help.

Now, outside of the university, based on my own experience, I think the barrier is the culture itself. We don’t have the culture of seeking out a psychologist or psychological care. Our culture is to seek out a doctor at most. And when they do, someone refers you to a psychiatrist, and they start thinking you’re crazy. So that’s not the case, it’s not popular. I’m not just talking about the price, but I’m talking about people knowing that they can have therapy. Therapy in our society is like this: the elite have therapy and we work here (Course coordinator, P28).

### Facilitators of help-seeking behavior

Data analysis allowed us to identify 12 (twelve) categories that illustrate the facilitators for seeking professional help, as detailed in Table 7. Health professionals added the following answers to the questionnaire: financial support from the institution, suffering a lot and really needing help, and damage caused by suffering and psychological distress (emotional pain). Some course coordinators mentioned that the university does not offer opportunities for students to seek help (16.66%), while others were unable to say whether there are facilitators (8.33%).

**Table 7:** Facilitators for professional help-seeking behavior according to students, health professionals, technical staff and undergraduate courses coordinators

Level	Facilitators	Study	Profession	Technical	Coord
Individual	Mental health literacy	15%	72.7%	---	---
	Emotional pain resulting from suffering and psychological disorder	---	9%	---	---
	Positive previous experience with therapy	15%	40.9%	---	---
	Previous positive experience with medical treatment	---	27.3%	---	---
Interpersonal	Social support from one’s immediate social network	15%	77.3%	---	---
	Having close friends who have sought treatment	10%	68.2%	40%	---
Organizational	Financial support from the university	---	4.5%	80%	8.3%
	Adequate infrastructure of health services at the university	---	---	20%	---
	Course offering on Happiness	---	---	---	8.3%
	Proximity to service (having a psychologist on campus)	---	---	20%	66.7%
	Knowing that financial aid exists	10%	---	---	---
	Use of humor and informal language in freshman reception activities	---	---	20%	---

Percentages do not add up to 100% because participants could mention more than one facilitator for help-seeking behavior

*Mental health literacy* category includes reports on understanding the signs and symptoms of mental disorders and the need to seek help from a mental health professional. These reports were found primarily among students who had previous experience with psychotherapy and those in healthcare courses.

The first time I went to the psychologist, I went alone, because I felt that I was different. I was more stressed, without patience (…). I didn’t have any problems, I said that I thought I was depressed (Student, P16).

The category *positive previous experience with psychotherapy* includes reports from students who had already undergone the psychotherapy process and who felt satisfied.

(…) the fact of seeking help was not difficult. When I was a teenager, I started therapy (…). (Student, P15).

The category social support from one’s immediate social network includes reports of having people close to them, such as parents, friends, romantic partners, and teachers who encouraged the student to seek treatment. In some cases, these people went with the students to the health service.

One day in class I said: “Oh my God, I can’t take it anymore, I want to die”. Then a teacher heard me, and he was the one who told me about the aid, who actually went after me. He was the one who took me to the student assistance center. Because when he called me to talk, he asked me if something was wrong. And I told him everything, right? And he took me, I didn’t know before (Student, P8).

The category of *having close friends who have sought treatment* includes reports of living with a person who underwent psychotherapy and who advises the student to seek help.

(…) also the issue of word of mouth. When you have a student, he comes to you and you listen to him and he gets feedback. He sees that it was a welcome, that it is confidential, that he can open up in that environment, he also tells other people. (…) And I saw that as I was attending, there were other students who came because the colleague said that he had already been to the psychologist (Technical-administrative employee, P37).

The category of *financial support of the institution* includes reports of financial aid offered by the educational institution that allows the student to pay for mental health treatment.

For me, it’s definitely the aid. The aid is a game changer, like. During the period I have a peak in service, during the period of public notices. So the aid made things much easier. It made things easier because the student knows that he will have money to get treatment, he will have access to this support, and it brought, what I noticed, is that the aid brought more serious cases (Technical-administrative employee, P36).

The category *adequate infrastructure of health services at the university* includes reports of improvements to the infrastructure of the sectors that offer health services, with spaces that are more accessible and comfortable.

We are in a better situation today, because two years ago we didn’t even have an individual service space. So it was much more complicated (Technical-administrative server, P36).

The category *course on happiness* includes reports on the “Pleasure and Suffering at the University module” offered to students of some undergraduate courses.

(…) the module aimed at promoting happiness (Course coordinator, P32).

The category *proximity to service (having a psychologist on campus)* covers reports regarding the Student Assistance Center (SAC) of each campus, where most of them have a psychologist on staff.

The university now has (referring to the student assistance center), which we can refer to. And the student has (…) extended service. (…). I think this helps a lot. (…) Because really, before we didn’t even know who to approach. (…) They help right away (Course coordinator, P31).

*Knowing that financial aid exists* category includes reports of students having information about financial aid, knowing that they can request it when they need it, and making mental health treatment something closer to everyday life, less stigmatized.

I knew there was financial aid. So, I knew about the existence of the aid and that made me feel more comfortable, saying: “Hmm, there is aid and other students are looking for it, so I can look for it too” (Student, P1).

The category *use of humor and informal language in freshman reception activities* includes reports of adjustments in language to create greater rapport with the student.

(…) one of the facilitators, at least in our experience (…) is the reception of freshmen. We managed to divide the reception of freshmen into the more technical part, and (…) an extremely playful, more fun, more relaxed talk, trying to speak more of their language. And this has made things much easier since we started doing this, because they feel that the service can welcome them in a language, in a way that is not so technical and that ‘someone will understand me’. People like us, who will understand us. This has made things much easier, it is very common after the reception of freshmen to have a *boom* in service, especially for incoming students. And then it creates a culture, I think it starts to create a culture among incoming students of accessibility and free access to service (Technical-administrative employee, P36).

### Social actors involved in help-seeking

Several social actors were involved in students’ help-seeking behavior. When questioned, 50% of students reported that they sought help on their own. Those who were referred reached the SAC or the university’s financial aid through: a friend/classmate, intimate partner, and professor, according to both students and health professionals, as can be seen in Table 8. Regarding other actors, health professionals added the following answer to the questionnaire: “Religious leaders make people suffer more” (5%). From the point of view of technical-administrative staff, even in the case of the student who sought the service on his own, he was previously encouraged by a professor, friend, classmate, and/or technical-administrative staff member (especially the course secretary). For most participants interviewed, friends or classmates are the main social actors involved in helping behavior.

**Table 8:** Social actors involved in help-seeking by students with suicidal ideation according to the students, health professionals and technical staff

How did you get into the service?	Social actors	Students	Health professionals	Technical staff	
Searched alone		50%	-	80%	
Forwarded by	friend / classmate	20%	95%	40%	60%
	teacher	10%	30%		80%
	intimate partner	15%	50%		
	technical-administrative	5%	-		20%
	country	-	5%		-
	other family members	-	5%	-	-
	community leaders	-	5%	-	-
	religious leaders	-	22.7%	-	-

## Discussion

This study analyzed the barriers, facilitators, and social actors involved in help-seeking behavior among students with suicidal ideation. The main finding was that a low perceived need for professional help and a tendency toward self-sufficiency constituted the strongest barriers, while mental health literacy and social support from one’s immediate social network emerged as consistent facilitators. These results are aligned with previous research (Czyz et al., 2013; De Luca et al., 2020; Downs et al., 2012; Eisenberg et al., 2012; Ibrahim et al., 2019; Lueck et al., 2020; Sagar-Ouriaghli et al., 2020; Tran et al., 2015) while advancing the field by integrating organizational and contextual dimensions seldom explored in studies from low- and middle-income countries. This expanded perspective highlights that help-seeking behavior is shaped not only by individual factors but also by institutional cultures and broader social inequalities.

In addition to structural inequalities, aspects related to gender and race/ethnicity may also shape students’ experiences of psychological distress and help-seeking. Given that most participants were women and many identified as Black or biracial, the findings may reflect experiences associated with minority stress, defined as the chronic psychological strain arising from exposure to prejudice, discrimination, and social marginalization (Meyer, 2003; Pascoe & Richman, 2009). In the Brazilian context, female students and students from racial minorities often face additional barriers in academic and institutional environments, including subtle forms of racism and gender bias, which can exacerbate mental health symptoms and feelings of isolation (Guerra et al., 2024; Oliveira et al., 2020). Recognizing these intersectional factors is essential for designing inclusive mental health strategies that address both individual vulnerability and systemic inequities.

The finding that many students reported a low perceived need for professional help may also be linked to limited mental health literacy, particularly regarding the recognition of symptoms and the available forms of psychological support (Jorm, 2012; Wei et al.,^2016^). Low mental health literacy can lead students to normalize emotional distress or to perceive it as a sign of weakness, thus reinforcing self-sufficiency and delaying help-seeking. Previous research indicates that improving mental health literacy among young adults increases their readiness to seek professional help and reduces stigma-related barriers (Gorczynski et al., 2017; Loureiro et al., 2013). These findings underscore the importance of integrating psychoeducational initiatives into university programs to foster early recognition of distress and promote a culture of care.

Although these general trends reveal the influence of contextual and structural conditions, it is also important to understand how personal beliefs and emotional bonds shape students’ willingness to seek support. At the individual level, participants reported difficulties in expressing emotions, fear of exposure, and feelings of shame and self-stigma, which delay or prevent help-seeking. These findings are consistent with both international (Downs et al., 2012; Ibrahim et al., 2019) and Brazilian literature (Falcão et al., 2021; Venturini & Goulart, 2016), evidencing the emotional and moral burden associated with mental health problems in competitive academic environments. Conversely, encouragement from peers, professors, and family members acted as a facilitator for seeking professional support, confirming that social connectedness is an important protective factor for mental health (Downs & Eisenberg, 2012).

Positive previous experiences with psychotherapy also proved relevant, whereas negative experiences – such as perceived lack of empathy, racial microaggression or cultural insensitivity – undermined trust in mental health professionals and reduced motivation to seek future help (Davis et al., 2018; Swift & Greenberg, 2012; Norcross & Lambert, 2018). These findings highlight the importance of ethical, culturally competent, and person-centered clinical practices within university mental health services.

At the interpersonal level, the dynamics of social networks played a crucial role in help-seeking behavior. The main barriers identified were insufficient social support and a preference for talking to friends rather than professionals. These results suggest that although peers often serve as an initial source of emotional support, they may inadvertently delay professional help-seeking when lacking the knowledge or confidence to refer someone appropriately (Czyz et al., 2013; Downs & Eisenberg, 2012). Conversely, encouragement from close individuals and having friends who had previously sought therapy demonstrate the positive contagion effect that supportive relationships can have on mental health behaviors (Ibrahim et al., 2019).

Friends, classmates, intimate partners, professors, and administrative staff thus emerge as central social actors who can facilitate or hinder access to professional care. Studies show that peer and faculty networks act as ‘gatekeepers’, identifying early signs of distress and facilitating referrals (Cecchin et al., 2022; Sagar-Ouriaghli et al., 2020; Drum & Denmark, 2012; Witt et al., 2019). Strengthening these bonds through training and institutional listening channels can enhance universities’ capacity to respond to psychological distress. Recent evidence indicates that peer and gatekeeper training programs increase the use of mental health services and reduce suicidal ideation (Cecchin et al., 2022; Cecchin et al., 2024; De Luca et al., 2020), reinforcing the strategic importance of supportive interpersonal environments within universities.

Beyond personal and relational factors, the institutional context decisively shapes how students experience and respond to psychological suffering. Organizational barriers such as understaffing, limited visibility of services, and bureaucratic procedures point to structural weaknesses in Brazilian universities, where mental health support programs are often underfunded and disconnected from the public health network (Araújo, 2020; Bleicher & Oliveira, 2016; Tavares et al., 2015). These findings highlight the need for integrated interventions that address both individual and institutional dimensions of university life.

At the organizational level, the main facilitators of help-seeking included the use of humor and informal language in student-focused activities – particularly during entry programs – and the availability of financial aid for psychological treatment. Such initiatives promote emotional accessibility and reduce the distance between students and institutional services. By adopting a more welcoming, inclusive, and student-centered communication style, universities can mitigate stigma and promote openness to discuss mental health issues (Sagar-Ouriaghli et al., 2020). The combination of financial support and relational engagement also contributes to students’ sense of belonging and trust in the institution, which are essential for psychosocial development (Chickering & Reisser, 1993; Drum & Denmark, 2012, Gacia Cecchin & Murta,^ 2025^). National surveys show that institutional belonging is associated with higher well-being and student engagement (Cecchin et al.,^2024^; Fórum Nacional de Pró-Reitores de Assuntos Estudantis, 2019). Thus, assistance policies, welcoming activities, and empathetic communication act as protective structures that can transform the university into a health-promoting setting. This approach aligns with the Okanagan Charter (World Health Organization, 2015), a global declaration that guides universities in integrating health promotion into all aspects of campus culture, emphasizing equity, well-being, and social connectedness.

From a critical social psychology perspective (Sawaia, 2014), student suffering cannot be understood without considering social inequalities and institutional dynamics that perpetuate exclusion and silence. Intersectional approaches (Crenshaw, 1989; Curiel, 2011) further highlight how gender, race, class, and sexuality intersect in shaping experiences of distress and access to care. It is also necessary to critically examine the centrality of outpatient care models in universities: although individual psychotherapy and clinical referrals remain essential, their predominance a reactive and medicalized logic. In line with the Okanagan Charter (World Health Organization, 2015), higher education institutions must adopt community-based, preventive, and intersectoral strategies focused on belonging, equity, and social justice.

These findings have practical implications for the development of university mental health programs. The methodological approach adopted in this study – integrating qualitative and quantitative data and engaging multiple institutional actors – proved effective and replicable for accurate needs assessment and co-development of contextually grounded solutions. These multiple informants can be implemented by other research aiming to design culturally sensitive and evidence-based mental health programs.

A practical application involves training university staff – such as professors, tutors, and administrative personnel – to recognize signs of distress and provide initial psychosocial support. Psychological First Aid (PFA) – based interventions have proven effective in non-clinical settings, offering structured guidance for active listening, emotional support, and safe referral to professional care (Hadlaczky et al., 2014; Maslowski et al.,^2019^; Shultz & Forbes, 2013; World Health Organization, 2011). Incorporating PFA principles into institutional training programs can strengthen universities’ ability to identify and respond early to student crises, especially in contexts with limited mental health resources.

This study has several limitations. First, qualitative data were not double-coded by independent raters, which may reduce interrater reliability. However, peer debriefing and member checking were implemented to enhance credibility. Additionally, although the sample size was adequate for an exploratory mixed-methods design, it limits the generalization of findings. Conducting the study in a single public university may also restrict transferability to other contexts. Self-report data may have been affected by recall bias or social desirability, and the lack of institutional records on help-seeking trajectories prevented more detailed correlational analyses.

It is also important to note that data collection took place during 2020, a period that overlapped with the COVID-19 pandemic. Although the study did not focus on pandemic-related variables, the exceptional social and academic conditions of that period may have influenced students’ mental health and help-seeking behavior. Future studies could further examine how the pandemic context affected these processes in university settings.

Future research should explore help-seeking behavior among different student populations – such as graduate students, students from private universities, and those from diverse cultural backgrounds – and examine longitudinally how institutional policies, stigma reduction campaigns, and peer interventions influence help-seeking over time. Experimental and implementation studies could also assess the impact of PFA-based training and other low-intensity interventions delivered by non-specialists. Collaborative and participatory approaches involving students, faculty, and mental health professionals are likely to generate more sustainable and inclusive institutional responses to psychological distress.

In summary, this discussion contributes to a contextual and socially engaged understanding of suicide prevention in higher education. It demonstrates how a multi-informant and mixed-methods approach can guide policy development and institutional decision-making, providing a foundation for interventions that move beyond individual-level solutions toward collective, structural, and culturally responsive transformation.

Overall, the results reinforce that mental health promotion and suicide prevention in higher education require a systemic approach that integrates individual, relational, organizational, and structural dimensions. The integration of qualitative and quantitative data, combined with input from multiple institutional actors, enabled a comprehensive understanding of the factors influencing help-seeking behavior and the identification of strategic points for intervention. By revealing how suicidal ideation is produced and sustained by institutional and sociocultural conditions, this research contributes to the fields of Social Psychology and Public Health by offering a contextualized and intersectoral interpretation of the phenomenon. Furthermore, it demonstrates the potential of low-cost strategies – such as the use of Psychological First Aid – to strengthen care networks and democratize access to psychological support. Thus, this study provides theoretical and practical insights for developing more equitable and sustainable institutional policies capable of transforming universities into spaces of belonging, solidarity, and life promotion.

## Conclusion

The main barrier to help-seeking behavior among university students with suicidal ideation is the low perceived need for professional help, often rooted in a tendency toward self-sufficiency, self-stigma, and emotional avoidance. Conversely, the most significant facilitator is mental health literacy, alongside encouragement from close individuals and previous positive experiences with psychotherapy. By adopting a mixed methods design and engaging multiple informants – including students, health professionals, administrative staff, and course coordinators – the research offers a nuanced and context-sensitive understanding of help-seeking behaviors. The findings underscore the urgent need for institutionally integrated, culturally competent, and stigma-sensitive interventions that extend beyond the individual to address organizational and systemic barriers. This study contributes valuable evidence to the field of university mental health by demonstrating that fostering a culture of early intervention, peer support, and health promotion is essential for effective suicide prevention. It provides a robust foundation for designing targeted programs that align with students lived experiences and institutional realities, particularly within the Brazilian context and other similar low-resource educational settings.

## Supplementary Information

Supplementary Material 1.

## Data Availability

Datasets used and/or analyzed during the current study are available from the corresponding author upon reasonable request.

## Abbreviations

DSM-5: Diagnostic and Statistical Manual of Mental Disorders, 5th edition
FICF: Free and Informed Consent Form
FIHE: Federal Institutions of Higher Education
SAC: Student Assistance Center
PROSA: Pro-Rectory of Student Affairs
PROUS: Pro-rectory of Undergraduate Studies

## Footnotes

1 This is a suicide prevention program whose name has been hidden so as not to identify the institution where the research was carried out.
