# Supplementary material for: Help-seeking behavior among college students with suicidal ideation: barriers, facilitators, and social actors
Source: Psicol Reflex Crit. 2026 Jan 22;39:4. doi: 10.1186/s41155-025-00374-x (PMC12824085; doi:10.1186/s41155-025-00374-x)
Supplement: Supplementary file 1 — Supplementary Material 1. [file 41155_2025_374_MOESM1_ESM.docx]

Additional file 1

**Table 1. Semi-structured interview and focus group guide**

| **Theme** | **Guiding question** | **Probes / Follow-ups** |
| --- | --- | --- |
| Barriers to help-seeking | What was it like for you to ask for help? | What made it difficult? How did you feel at that moment? |
| Facilitators to help-seeking | What was it like for you to ask for help? | What made it easy? What factor makes it easier for you to feel motivated to seek help from a health professional? |
| Social Actors | Who encouraged you to seek help? | How did they support you? Did that make a difference? |
